# Challenges in Treating Psychosis Amid Homelessness: A Case of an Undocumented Migrant in Spain

**DOI:** 10.1192/j.eurpsy.2026.11635

**Published:** 2026-07-31

**Authors:** M. Ligero Argudo, I. M. Peso Navarro, C. García Cerdán, C. Payo Rodríguez, R. K. González Bolaños, J. I. de la Iglesia, R. García-Andrade

**Affiliations:** 1 Psychiatry, Hospital Clínico de Salamanca, Salamanca; 2 ECASAM, Psychiatry, Hospital Clínico San Carlos, Madrid, Spain

## Abstract

**Introduction:**

Homelessnes represents a growing social and health emergency in Spain. According to the 2022 INE survey, 28’500 people are homeless, though NGOs estimate around 37’000-42’300 individuals. In the last decade, homelessness has increased by 25%, with only 28’00 shelter places recorded. Residential exclusion (overcrowding, insecure housing, informal settlements…) affects 18% of the population.

Groups mainy affected by this are vulnerable populations such as undocumented migrants. Recent resources show migrants have 7’5 more risk of homelessness than Spanish nationals. These conditions exacerbate barriers to healthcare access and standard treatment.

**Objectives:**

To describe a clinical case of severe psychotic disorder with resistance to usual treatment and severe social vulnerability.

To assess the impact of unstable living conditions on the clinical management of mental health disorders and, in special, treatment-resistant psychosis.

**Methods:**

A retrospective descriptive studio is conducted based on a clinical case admitted in an Acute Hospitalization Unit in Madrid, Spain and the literature available about homelessness and mental health conditions.

Clinical case: A young adult male, approximately 23-29 years, is brought to the hospital after jumping from a bridge in relation to mystical-religious delusions. The patient is undocumented, with unknown origin and no known relatives. He is not registered in the healthcare system and uses multiple pseudonyms. In Madrid, the Mental Health Street Team, which provides care for patients in social exclusionwho cannot access the standard healthcare network, was notified. Patient history was obtained from previous encounters and self-report, indicating he has been in Spain for approximately one year, with prior stays and hospitalizations in France and Germany. Clinical assessment focused on psychotic symptoms, including grandiose and religious–mystical delusions.

Following stabilization, with improved behavior and reduced interference, he was discharged to a residential resource. Since then, he has been admitted to the hospital in two more occasions due to conductual disturbances.

**Results:**

The patient exhibited psychotic symptoms resistant to both typical and atypical antipsychotics. The usual treatment of choice in these cases, clozapine, could not be initiated due to the imposibility of guaranteeing adequate adherence and monitoring.

**Conclusions:**

This case illustrates how homelessness in Spain critically limits access to effective treatment for severe psychotic disorders, especially in undocumented individuals. Strengthened coordination between healthcare and social services is essential to safeguard the right to health for socially excluded populations. Housing-focused strategies and adaptable healthcare resources are crucial to addressing the needs of marginalized groups.

**Disclosure of Interest:**

None Declared

